# Supplementary material for: Gliding motility of the diatom Craspedostauros australis coincides with the intracellular movement of raphid-specific myosins
Source: Commun Biol. 2024 Sep 23;7:1187. doi: 10.1038/s42003-024-06889-w (PMC11420354; doi:10.1038/s42003-024-06889-w)
Supplement: Supplementary file 2 — Supplementay Information [file 42003_2024_6889_MOESM2_ESM.pdf]

Gliding motility of the diatom *Craspedostauros australis* coincides with the intracellular movement of raphid-specific myosins

Metin Gabriel Davutoglu[1], Veikko F. Geyer[1], Lukas Niese[1], Johannes Müller[2], Marcelo Leomil Zoccoler[2], Valeria Sabatino[1], Robert Haase[2], Nils Kröger[1,2,3], Stefan Diez[1,2, 4]*, Nicole Poulsen[1]*

[1] B CUBE - Center for Molecular Bioengineering, TUD Dresden University of Technology, 01307 Dresden, Germany

[2] Cluster of Excellence Physics of Life, TUD Dresden University of Technology, 01062 Dresden, Germany

[3] Faculty of Chemistry and Food Chemistry, TUD Dresden University of Technology, 01062 Dresden, Germany

[4] Max Planck Institute of Molecular Cell Biology and Genetics, 01307 Dresden, Germany

*correspondence to: stefan.diez@tu-dresden.de, nicole.poulsen@tu-dresden.de

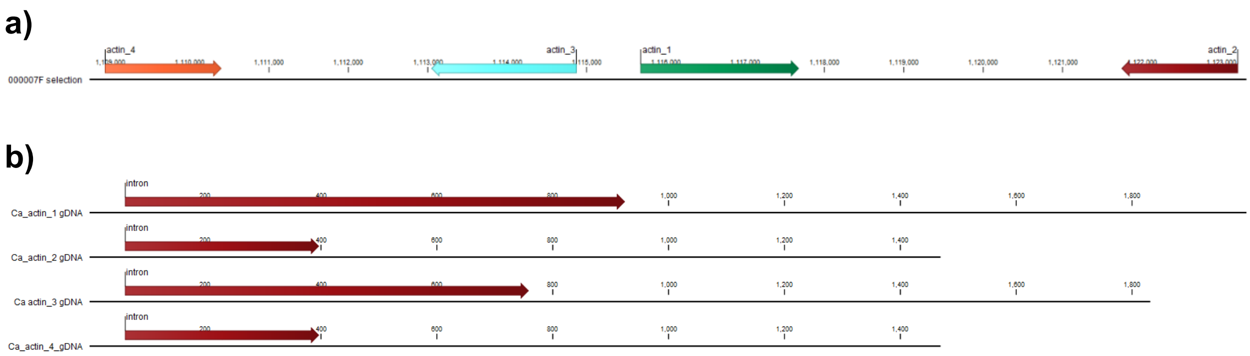

Supplementary Figure 1: (a) Genomic arrangement of the four *C. australis* actin genes on the Pacbio Genome assembly contig 7F[1] **(b)** Gene structure of the four *C. australis* actin genes showing the position of the intron.

```
actin_1      MSDDEDIAALVIDNGSGMCKAGFAGDDAPRSVFPSLIGRARQPGIMVGMEQRDAYVGDEA      60
actin_2      MSDDEDIAALVIDNGSGMCKAGFAGDDAPRSVFPSLIGRARQPGIMVGMEQRDAYVGDEA      60
actin_3      MSDDEDIAALVIDNGSGMCKAGFAGDDAPRSVFPSLIGRARQPGIMVGMEQRDAYVGDEA      60
actin_4      MSDDEDIAALVIDNGSGMCKAGFAGDDAPRSVFPSLIGRARQPGIMVGMEQRDAYVGDEA      60
             ************************************************************

actin_1      QAKRGVLTLKYPIEHGIVTNWDDMEKIWHHTFYNELRVAPEAHPVLLTEAPQNPKANRER      120
actin_2      QAKRGVLTLKYPIEHGIVTNWDDMEKIWHHTFYNELRVAPEAHPVLLTEAPQNPKANRER      120
actin_3      QAKRGVLTLKYPIEHGIVTNWDDMEKIWHHTFYNELRVAPEAHPVLLTEAPQNPKANRER      120
actin_4      QAKRGVLTLKYPIEHGIVTNWDDMEKIWHHTFYNELRVAPEAHPVLLTEAPQNPKANRER      120
             ************************************************************

actin_1      MTQIMFETFNVPAMYVNIQAVLSLYASGRTTGCVLDSGDGVTHTVPIYEGYALPHAVIRL      180
actin_2      MTQIMFETFNVPAMYVNIQAVLSLYASGRTTGCVLDSGDGVTHTVPIYEGYALPHAVIRL      180
actin_3      MTQIMFETFNVPAMYVNIQAVLSLYASGRTTGCVLDSGDGVTHTVPIYEGYALPHAVIRL      180
actin_4      MTQIMFETFNVPAMYVNIQAVLSLYASGRTTGCVLDSGDGVTHTVPIYEGYALPHAVIRL      180
             ************************************************************

actin_1      DLAGRDLTEYLMKILTERGYSLTTTAEREIVRDIKESLCFVAVDFEEEMKKAAESSALEK      240
actin_2      DLAGRDLTEYLMKILTERGYSLTTTAEREIVRDIKESLCFVAVDFEEEMKKAAESSALEK      240
actin_3      DLAGRDLTEYLMKILTERGYSLTTTAEREIVRDIKESLCFVAVDFEEEMKKAAESSALEK      240
actin_4      DLAGRDLTEYLMKILTERGYSLTTTAEREIVRDIKESLCFVAVDFEEEMKKAAESSALEK      240
             ************************************************************

actin_1      SFELPDGNIIVIGNERFRCPEVLFQPNLTGLEMDGIGDSTFQTIMKCDVDIRKDLYANIV      300
actin_2      SFELPDGNIIVIGNERFRCPEVLFQPNLTGLEMDGVGDSTFQTIMKCDVDIRKDLYANIV      300
actin_3      SFELPDGNIIVIGNERFRCPEVLFQPNLTGLEMDGVGDSTFQTIMKCDVDIRKDLYANIV      300
actin_4      SFELPDGNIIVIGNERFRCPEVLFQPNLTGLEMDGVGDSTFQTIMKCDVDIRKDLYANIV      300
             ***********************************:************************

actin_1      LSGGTTMFPGISERMSKEITALAPASIKVKIVAPPERKYSVWIGGSILASLSTFQSMWIS      360
actin_2      LSGGTTMFPGISERMSKEITALAPASIKVKIVAPPERKYSVWIGGSILASLSTFQSMWIS      360
actin_3      LSGGTTMFPGISERMSKEITALAPASIKVKIVAPPERKYSVWIGGSILASLSTFQSMWIS      360
actin_4      LSGGTTMFPGISERMSKEITALAPASIKVKIVAPPERKYSVWIGGSILASLSTFQSMWIS      360
             ************************************************************

actin_1      KEEYDESGPSIVHRKCF*          377
actin_2      KEEYDESGPSIVHRKCF*          377
actin_3      KEEYDESGPSIVHRKCF*          377
actin_4      KEEYDESGPSIVHRKCF*          377
             ******************
```

Supplementary Figure 2: Clustal Omega alignment of the four *C. australis* actin protein sequences showing a single amino acid exchange. Identical amino acid residues are highlighted in grey and indicated with an asterisk (*), a : (colon) indicates conservation between groups of strongly similar properties.

```
actin_4_cDNA      ATGTCTGACGATGAAGATATCGCCGCCCCTTGTCATCGACAACGGTTCCGGAATGTGCAAA      60
actin_3_cDNA      ATGTCTGACGACGAAGATATCGCCGCCCCTTGTCATCGACAACGGTTCCGGAATGTGCAAG      60
actin_1_cDNA      ATGTCTGACGACGAAGATATCGCCGCCCCTTGTCATCGACAACGGTTCCGGAATGTGCAAA      60
actin_2_cDNA      ATGTCTGACGATGAAGATATCGCCGCCCCTTGTCATCGACAACGGTTCCGGAATGTGCAAA      60
                  *********** ***********************************************

actin_4_cDNA      GCCGGATTCGCTGGAGATGACGCCCCACGCTCCGTCTTCCCATCCCTCATCGGACGTGCC      120
actin_3_cDNA      GCCGGATTCGCTGGAGATGACGCCCCACGCTCCGTCTTCCCATCCCTCATCGGACGTGCC      120
actin_1_cDNA      GCCGGATTCGCTGGAGATGACGCCCCACGCTCCGTCTTCCCATCCCTCATCGGACGTGCC      120
actin_2_cDNA      GCCGGATTCGCTGGAGATGACGCCCCACGCTCCGTCTTCCCATCCCTCATCGGACGTGCC      120
                  ************************************************************

actin_4_cDNA      CGCCAGCCAGGTATCATGGTTGGTATGGAGCAGCGTGACGCATACGTCGGTGACGAGGCA      180
actin_3_cDNA      CGCCAGCCAGGTATCATGGTTGGTATGGAGCAGCGTGACGCATACGTCGGTGATGAGGCA      180
actin_1_cDNA      CGCCAGCCAGGTATCATGGTCGGTATGGAGCAGCGTGACGCATACGTCGGTGACGAGGCA      180
actin_2_cDNA      CGCCAGCCAGGTATCATGGTCGGTATGGAGCAGCGTGACGCATACGTCGGTGACGAGGCA      180
                  ******************** ******************************** ******

actin_4_cDNA      CAGGCCAAGCGTGGTGTCCTCACACTGAAGTACCCAATCGAGCACGGCATCGTCACCAAC      240
actin_3_cDNA      CAGGCCAAGCGTGGTGTCCTCACACTGAAGTACCCAATCGAGCACGGTATCGTCACCAAC      240
actin_1_cDNA      CAGGCCAAGCGTGGTGTCCTCACACTGAAGTACCCAATCGAGCACGGTATCGTCACCAAC      240
actin_2_cDNA      CAGGCCAAGCGTGGTGTCCTCACACTGAAGTACCCAATCGAGCACGGTATCGTCACCAAC      240
                  *********************************************** ************

actin_4_cDNA      TGGGACGATATGGAGAAGATCTGGCATCACACCTTCTACAACGAGCTCCGTGTCGCCCCA      300
actin_3_cDNA      TGGGACGATATGGAGAAGATCTGGCATCACACCTTCTACAACGAGCTCCGTGTCGCCCCA      300
actin_1_cDNA      TGGGACGATATGGAGAAGATCTGGCATCACACCTTCTACAACGAGCTCCGTGTCGCCCCA      300
actin_2_cDNA      TGGGACGATATGGAGAAGATCTGGCATCACACCTTCTACAACGAGCTCCGTGTCGCCCCA      300
                  ************************************************************

actin_4_cDNA      GAGGCCCACCCAGTCCTGCTCACCGAGGCCCCACAGAACCCCAAGGCCAACCGCGAACGT      360
actin_3_cDNA      GAGGCCCACCCAGTCCTGCTCACCGAGGCCCCACAGAACCCCAAGGCCAACCGCGAACGT      360
actin_1_cDNA      GAGGCCCACCCAGTCCTGCTCACCGAGGCCCCACAGAACCCCAAGGCCAACCGCGAGCGT      360
actin_2_cDNA      GAGGCCCACCCAGTCCTGCTCACCGAGGCCCCACAGAACCCCAAGGCCAACCGCGAGCGT      360
                  ******************************************************** ***

actin_4_cDNA      ATGACCCAGATCATGTTCGAGACCTTCAACGTCCCGGCCATGTACGTCAACATCCAGGCC      420
actin_3_cDNA      ATGACCCAGATCATGTTCGAGACCTTCAACGTCCCGGCCATGTACGTCAACATCCAGGCC      420
actin_1_cDNA      ATGACCCAGATCATGTTCGAGACCTTCAACGTCCCGGCCATGTACGTCAACATCCAAGCC      420
actin_2_cDNA      ATGACCCAGATCATGTTCGAGACCTTCAACGTCCCGGCCATGTACGTCAACATCCAAGCC      420
                  ******************************************************** ***

actin_4_cDNA      GTCCTCTCCCTGTACGCCTCTGGACGTACCACCGGATGCGTGCTGGACTCCGGTGATGGT      480
actin_3_cDNA      GTCCTCTCCCTGTACGCCTCCGGACGTACCACCGGATGCGTGCTGGACTCCGGTGATGGT      480
actin_1_cDNA      GTCCTCTCCCTGTACGCCTCTGGACGTACCACCGGATGCGTGCTGGACTCCGGTGATGGT      480
actin_2_cDNA      GTCCTCTCCCTGTACGCCTCCGGACGTACCACCGGATGCGTGCTGGACTCCGGTGATGGT      480
                  ******************** ***************************************

actin_4_cDNA      GTGACACACACTGTCCCAATCTACGAGGGATACGCCCTTCCACACGCCGTCATCCGTCTC      540
actin_3_cDNA      GTGACACACACCGTTCCAATCTACGAGGGATACGCTCTTCCACACGCCGTCATCCGTCTC      540
actin_1_cDNA      GTGACACACACCGTTCCAATCTATGAGGGATACGCCCTTCCACACGCCGTCATCCGTCTC      540
actin_2_cDNA      GTGACACACACCGTCCCAATCTATGAGGGATACGCTCTTCCACACGCCGTCATCCGTCTC      540
                  *********** ** ******** *********** ************************

actin_4_cDNA      GATTTGGCCGGACGTGACCTGACCGAGTACCTGATGAAGATCCTCACAGAGCGTGGTTAC      600
actin_3_cDNA      GATTTGGCCGGACGTGACCTGACCGAGTACCTGATGAAGATCCTCACAGAGCGTGGTTAC      600
actin_1_cDNA      GATTTGGCCGGACGTGACCTGACCGAGTACCTGATGAAGATCCTCACAGAGCGTGGTTAC      600
actin_2_cDNA      GATTTGGCCGGACGTGACCTGACCGAGTACCTGATGAAGATCCTCACAGAGCGTGGTTAC      600
                  ************************************************************

actin_4_cDNA      TCGCTGACAACCACCGCCGAGCGCGAAATCGTCCGTGACATCAAGGAGAGTCTCTGCTTC      660
actin_3_cDNA      TCGCTGACAACCACCGCCGAGCGCGAAATCGTCCGTGACATCAAGGAGAGTCTCTGCTTC      660
actin_1_cDNA      TCGCTGACAACCACCGCCGAGCGCGAAATCGTCCGTGACATCAAGGAGAGTCTCTGCTTC      660
actin_2_cDNA      TCGCTGACAACCACCGCCGAGCGCGAAATCGTCCGTGACATCAAGGAGAGTCTCTGCTTC      660
                  ************************************************************

actin_4_cDNA      GTCGCCGTCGACTTCGAGGAGGAGATGAAGAAGGCCGCTGAGTCTTCTGCTCTTGAGAAG      720
actin_3_cDNA      GTCGCCGTCGACTTCGAGGAGGAGATGAAGAAGGCCGCTGAGTCTTCTGCTCTTGAGAAG      720
actin_1_cDNA      GTCGCCGTCGACTTCGAGGAGGAGATGAAGAAGGCCGCTGAGTCTTCTGCTCTTGAGAAG      720
actin_2_cDNA      GTCGCCGTCGACTTCGAGGAGGAGATGAAGAAGGCCGCTGAGTCTTCTGCTCTTGAGAAG      720
                  ************************************************************
```

```
actin_4_cDNA      TCTTTCGAGCTTCCAGATGGTAACATCATCGTGATTGGAAACGAGCGCTTCCGTTGCCCA      780
actin_3_cDNA      TCTTTCGAGCTTCCAGATGGTAACATCATCGTGATTGGAAACGAGCGCTTCCGTTGCCCA      780
actin_1_cDNA      TCTTTCGAGCTTCCAGATGGTAACATCATCGTGATTGGAAACGAGCGCTTCCGTTGCCCA      780
actin_2_cDNA      TCTTTCGAGCTTCCAGATGGTAACATCATCGTGATTGGAAACGAGCGCTTCCGTTGCCCA      780
                  ************************************************************

actin_4_cDNA      GAGGTCCTGTTCCAGCCTAACTTGACTGGTCTCGAAATGGACGGTGTTGGCGACTCCACC      840
actin_3_cDNA      GAGGTCCTGTTCCAGCCTAACTTGACTGGTCTCGAAATGGACGGTGTTGGCGACTCCACC      840
actin_1_cDNA      GAGGTCCTGTTCCAGCCTAACTTGACTGGTCTCGAAATGGACGGTATTGGCGACTCCACC      840
actin_2_cDNA      GAGGTCCTGTTCCAGCCTAACTTGACTGGTCTCGAAATGGACGGTGTTGGCGACTCCACC      840
                  ********************************************* **************

actin_4_cDNA      TTCCAGACCATCATGAAGTGCGATGTCGATATCCGCAAGGATCTGTACGCCAACATCGTC      900
actin_3_cDNA      TTCCAGACCATCATGAAGTGCGATGTCGATATCCGCAAGGATCTGTACGCCAACATCGTC      900
actin_1_cDNA      TTCCAGACCATCATGAAGTGTGATGTCGATATCCGCAAGGATCTGTACGCCAACATCGTC      900
actin_2_cDNA      TTCCAGACCATCATGAAGTGCGATGTCGATATCCGCAAGGACCTGTACGCCAACATCGTC      900
                  ******************** ******************** ******************

actin_4_cDNA      CTTTCTGGAGGAACCACCATGTTCCCAGGAATCAGCGAGCGCATGTCGAAGGAGATCACC      960
actin_3_cDNA      CTTTCTGGAGGAACCACCATGTTCCCAGGAATTAGCGAGCGCATGTCGAAGGAGATCACC      960
actin_1_cDNA      CTTTCTGGAGGAACCACCATGTTCCCAGGAATTAGCGAGCGCATGTCGAAGGAGATCACC      960
actin_2_cDNA      CTTTCTGGAGGAACCACCATGTTCCCAGGAATCAGCGAGCGCATGTCGAAGGAGATCACC      960
                  ******************************** ***************************

actin_4_cDNA      GCCCTCGCCCCAGCCTCCATCAAGGTCAAGATCGTGGCCCCACCGGAGCGAAAGTACTCC      1020
actin_3_cDNA      GCCCTCGCCCCAGCCTCCATCAAGGTCAAGATCGTGGCCCCACCGGAGCGAAAGTACTCC      1020
actin_1_cDNA      GCCCTCGCCCCAGCCTCCATCAAGGTCAAGATCGTGGCCCCACCGGAGCGAAAGTACTCC      1020
actin_2_cDNA      GCCCTCGCCCCAGCCTCCATCAAGGTCAAGATCGTGGCCCCACCGGAGCGAAAGTACTCC      1020
                  ************************************************************

actin_4_cDNA      GTGTGGATCGGAGGATCCATCCTCGCTTCCCTGTCTACATTCCAGAGCATGTGGATCAGC      1080
actin_3_cDNA      GTGTGGATCGGAGGATCCATCCTCGCTTCCCTGTCTACATTCCAGAGCATGTGGATCAGC      1080
actin_1_cDNA      GTGTGGATCGGAGGATCCATCCTCGCTTCCCTGTCTACATTCCAGAGCATGTGGATCAGC      1080
actin_2_cDNA      GTGTGGATCGGAGGATCCATCCTCGCTTCCCTGTCTACATTCCAGAGCATGTGGATCAGC      1080
                  ************************************************************

actin_4_cDNA      AAGGAGGAGTACGACGAGTCCGGCCCATCCATCGTCCACCGCAAGTGCTTCTAA    1134
actin_3_cDNA      AAGGAGGAGTACGACGAGTCCGGTCCATCCATCGTCCACCGCAAGTGCTTCTAA    1134
actin_1_cDNA      AAGGAGGAGTACGACGAGTCCGGTCCATCCATCGTCCACCGCAAGTGCTTCTAA    1134
actin_2_cDNA      AAGGAGGAGTACGACGAGTCCGGTCCATCCATCGTCCATCGCAAGTGCTTCTAA    1134
                  *********************** ************** ***************
```

Supplementary Figure 3: Clustal Omega alignment of the four *C. australis* mRNA sequences. Identical nucleotides are highlighted in grey and indicated with an asterisk (*).

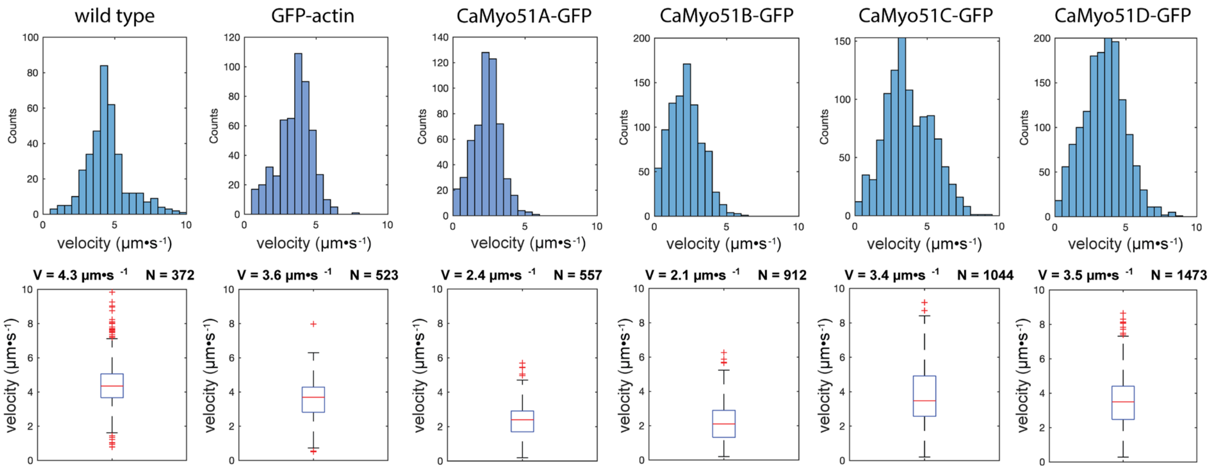

Supplementary Figure 4: Cell motility analysis of *C. australis* cell lines. Histograms (upper panels) and corresponding box plots (lower panels) showing the distribution of cell velocities in populations of wild type *C. australis* (far left) and *C. australis* cell lines expressing actin or myosins as GFP fusion proteins used for TIRFM analyses of actin and myosin dynamics. In the box plots, the red center lines denote the median (50th percentile) values, with the blue boxes having lower and upper bounds of the 25th and 75th, respectively. The black whiskers indicate the 5th and 95th percentiles, and outlier values outside of this range are indicated by red plus signs. N represents the number of investigated cells.

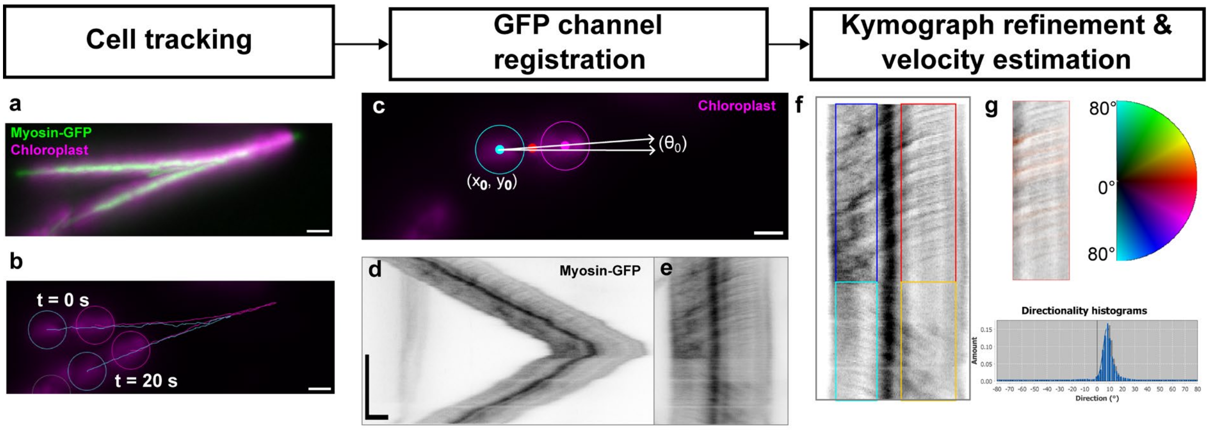

Supplementary Figure 5: Image analysis pipeline. a Maximum projection of a 20 s dual-channel movie of a gliding cell. **b** Overlay of tracks generated using TrackMate using the chloroplast autofluorescence signal in the far red optical spectrum. Chloroplast positions (circles) are shown only for the first and last frames. **c** Chloroplast positions for each frame were used to determine the position of the midpoint between the two chloroplasts (red dot) and the angle of the cell relative to a horizontal origin. **d, e** Kymographs of myosin-GFP signals (in black) generated by reslicing and projecting each row of pixels of the image before (**d**) and after (**e**) registration using displacement and angle values. **f** Refined kymograph after performing a Fast-Fourier-Transformation, masking horizontal traces, and inverting the Fourier transformation. Indigo, red, cyan and yellow boxes indicate regions-of-interest (ROIs) used for directionality measurements. **g** Orientation map showing the preferred orientation of the myosin-GFP signals in the red ROI shown in **f** (top left), color wheel showing gradient of colors used to indicate angle (top right) and histogram of estimated angles generated by the directionality plugin in Fiji. Scale bars – horizontal 5 µm, vertical 5 s.

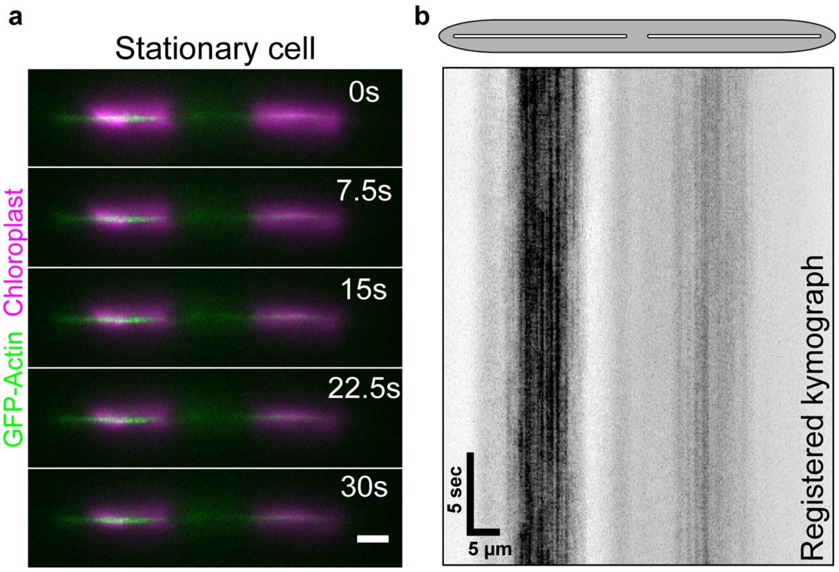

Supplementary Figure 6: TIRFM based kymograph analysis of GFP-actin in stationary cells. a Montage generated for dual-channel TIRFM data showing the positions of a stationary cell at 7.5 s intervals (GFP-actin in green and chloroplast autofluorescence in magenta, generated from Supplementary Movie S2). **b** Kymograph analysis of GFP-actin movement. GFP-channel data from the **Supplementary Movie S2** shown in **a** was registered using cell tracking data (GFP-actin in black). Gray ellipse and white slits above kymograph approximate the positions of the cell body and raphe openings, respectively. (Scale bars: horizontal = 5 µm, vertical = 5 s)

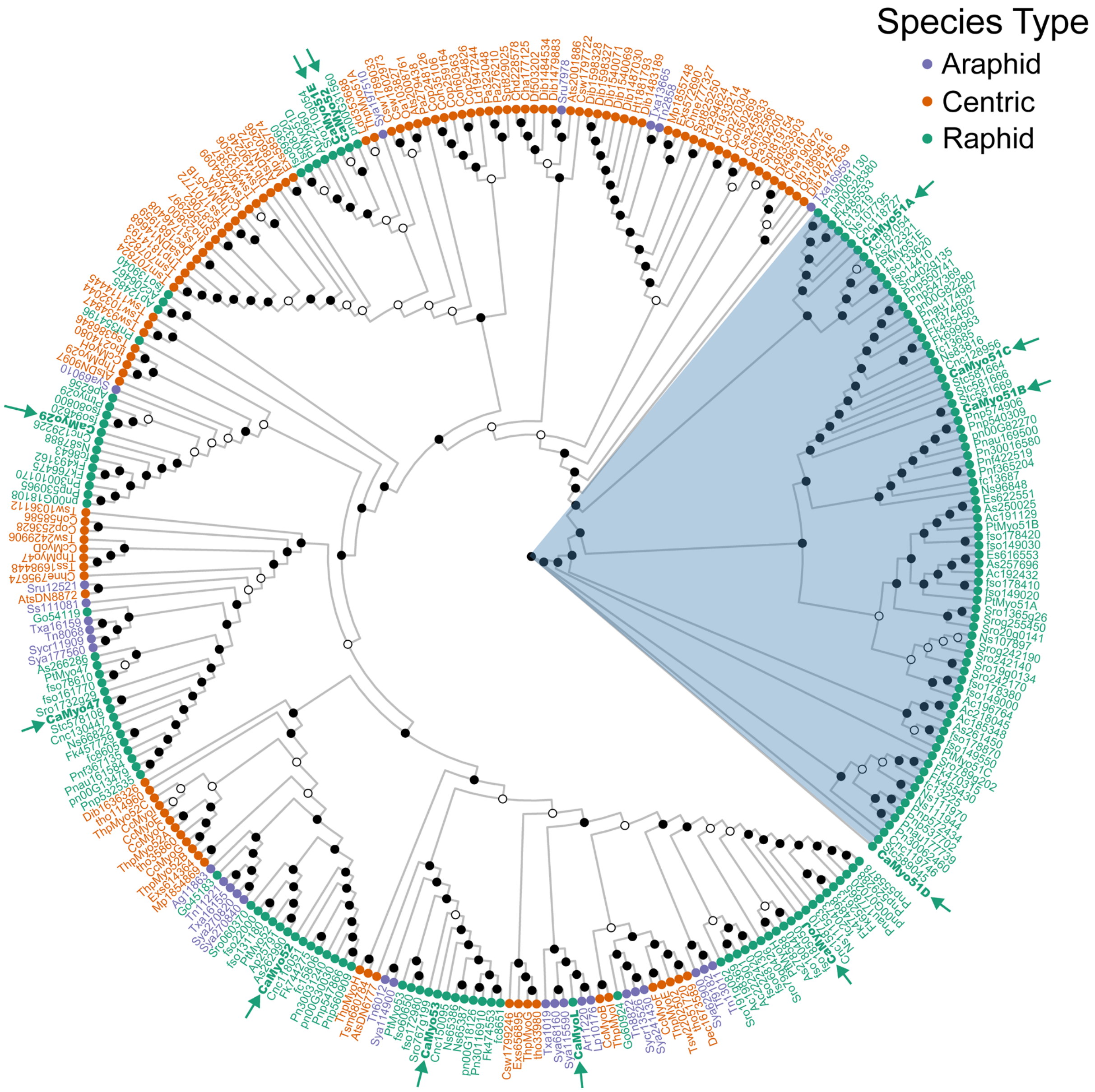

Supplementary Figure 7: Phylogenomic analysis of diatom myosins. Cladogram of 309 diatom myosins. The position of *C. australis* myosins (CaMyoX, 12 sequences) are in bold and labeled with a green arrow. A large clade containing only raphid diatom myosins, including the four *C. australis* myosins investigated in this study (CaMyo51A-D) is highlighted in blue. Node tips are color coded by species type. Internal nodes indicate Ufboot bootstrap scores of <95 (hollow nodes) or ≥95 (solid nodes). Species name are abbreviated as follows as the full sequence names can be found in Supplementary Table 1. *Amphora coffeaeformis* (Ac); *Asterionellopsis glacialis* (Ag); *Amphiprora paludosa* (Ap) *Astrosyne radiata* (Ar); *Amphiprora sp* (As); *Attheya septentrionalis* (Ats); *Craspedostauros australis* (Ca); *Cyclotella cryptica* (Cc); *Chaetoceros affinis* (Cha); *Chaetoceros debilis* (Chd); *Chaetoceros neogracile* (Chne); Cylindrotheca closterium (Cnc); *Corethron hystrix* (Coh); *Corethron pennatum* (Cop); *Coscinodiscus wailesii* (Csw); *Detonula confervacea* (Dec); *Dactyliosolen fragilissimus* (Df); *Ditylum brightwellii* (Dib); *Entomoneis sp* (Es); *Extubocellulus spinifer* (Exs); *Fragilariopsis*

cylindrus (Fc); *Fragilariopsis kerguelensis* (Fk); *Fistulifera solaris* (Fso); *Grammatophora oceanica* (Go); *Helicotheca tamesis* (Ht); *Leptocylindrus danicus* (Ld); *Leptocylindrus danicusvar danicus* (Ldd); *Licmophora paradoxa* (Lp); *Minutocellus polymorphus*(Mp); *Nitzschia sp* (Ns); *Odontella aurita* (Oa); *Proboscia alata* (Pa); *Pseudo-nitzschia multiseries* (Pn); *Pseudo-nitzschia australis* (Pnau); *Pseudo-nitzschia fraudulenta* (Pnf); *Pseudo-nitzschia pungens* (PnP); *Phaeodactylum tricornutum* (Pt); *Skeletonema costatum* (Sc); *Skeletonema menzelii* (Sm); *Stephanopyxis turris* (Spt); *Seminavis robusta* (Sro); *Striatella unipunctata* (Sru); *Staurosira sp.* (Ss); *Stauroneis constricta* (Stc); *Synedro acus* (Sya); *Synedropsis cf recta* (Sycr); *Thalassionema frauenfeldii* (Tf); *Thalassiosira pseudonana* (Thp); *Thalassionema nitzschioides* (Tn); *Thalassiosira antarctica* (Tsa); *Thalassiosira gravida* (Tsg); *Thalassiosira miniscula* (Tsm); *Thalassiosira sp.* (Tss); *Thalassiosira oceanica* (Tho); *Thalassiosira weissflogii* (Tsw); *Thalassiothrix antarctica* (Txa)

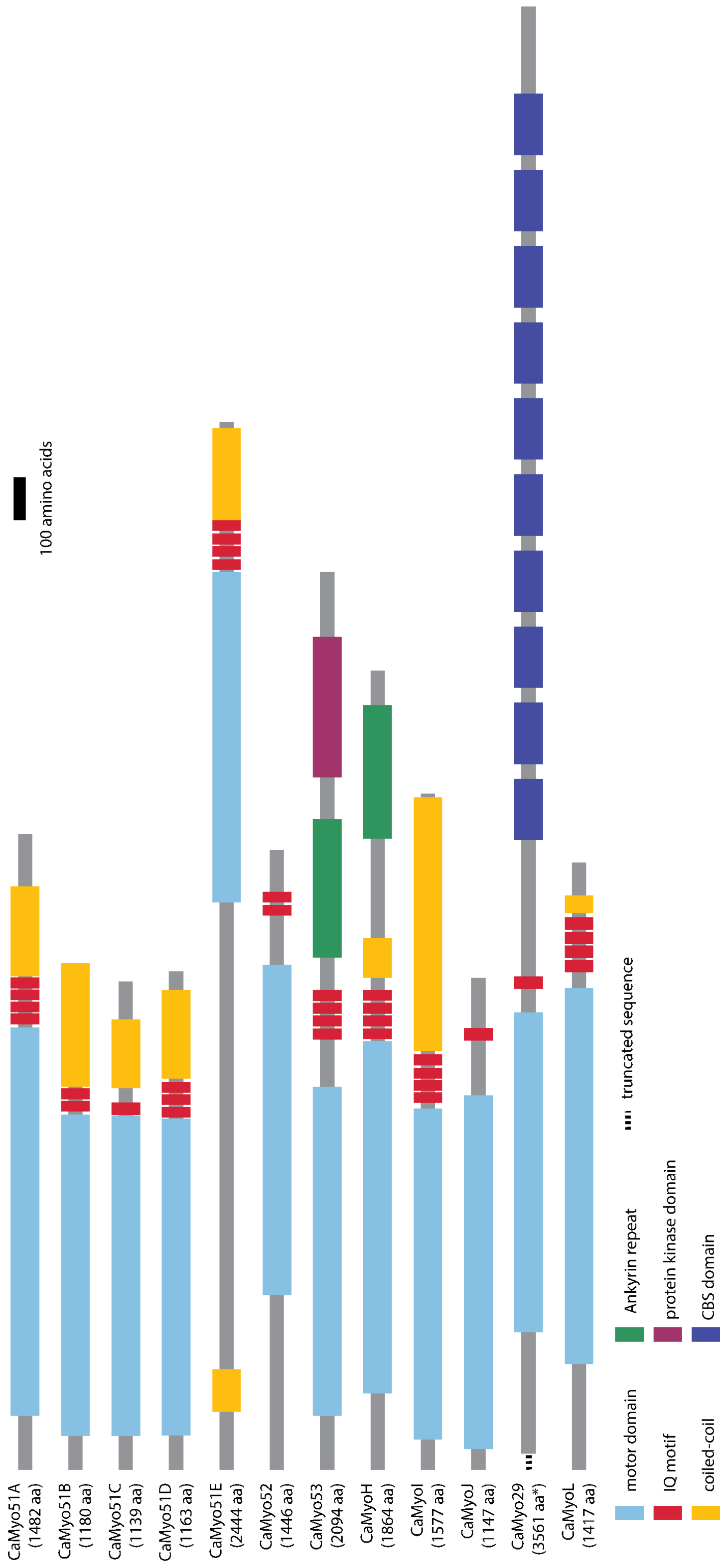

Supplementary Figure 8. Schematic of twelve predicted *C. australis* myosins

```
CaMyo51A       LHEAAILYNLKARHV-QALPYTRTGDIVIAVNPYQWFHVLYSHDNQNTYSEALIWKNRSN  59
CaMyo51D       LHEAAILYNLKERHC-SAKPYTRVSDIVIAVNPYQWLTDLYTEKVRLYYAQKLVWEQSE-  58
CaMyo51B       LHEAAILYNLKERHL-NNHPYTRTGDIVIAVNPFQWFKEIYTEKVRARFANKLVWEDHE-  58
CaMyo51C       LHEPAILFNLKERHG-SGKPYTRTGDIIIAVNPFQWFKEIYTEKVRNRYARILVWENVD-  58
CeMyo5         LHEPAVLHNLQVRFVKGSSIYTYCGIVLVAINPYADCSHIYGEEIIQVYRGA--------  52
DdMyo1E        ITENAFIENLTMRHK-SDNIYTYIGDVVISTNPFKNL-NIYKESDIKAYNGR--------  50
HsMhc2         LHEPAVLYNLKERYA-AWMIYTYSGLFCVTVNPYKWL-PVYKPEVVTAYRGK--------  50
OcMhc1         LHEPAVLYNLKEXYA-AWMIYTYSGLFCVTVNPYKWL-PVYNAEVVTAYRGK--------  50
               : * *.: **   .      **  . . :: **:     :*  .    :

CaMyo51A       ITKRKSTSKPNSNDNSNSNNSNNNQTKSQTQPQSQGEGASPTADISDPRNGLVPHVYEAS  119
CaMyo51D       ---------------------------------------------QDPRQGLPPHIYEVS  73
CaMyo51B       ---------------------------------------------GDARVLVEPHVYEAS  73
CaMyo51C       ---------------------------------------------GDQRSQVEPHVYETS  73
CeMyo5         ---------------------------------------------GKSAREMDPHIFAVA  67
DdMyo1E        -----------------------------------------------YKYEMPPHIYALA  63
HsMhc2         -----------------------------------------------KRQEAPPHIFSIS  63
OcMhc1         -----------------------------------------------KRQEAPPHIFSIS  63
                                                                    **::  :

                                   P-loop
CaMyo51A       ALAYQGLAARGDDQSILVSGESGAGKTETVKICMSHLASIQNLTPKQRQKQRVLNAAVSS  179
CaMyo51D       SLCYRGLAYEGVNQSILVSGESGAGKTETVKICMNHIASVQKDPTAV-------------  120
CaMyo51B       SLAYKGLAFEGSDQSILVSGESGAGKTETVKICMNHIASVQRGPTSD-------------  120
CaMyo51C       ALAYNGLAFGGNDQSILVSGESGAGKTETVKICMNHIASCQQGPTAK-------------  120
CeMyo5         EEAHFDMGAFGKSQSIIVSGESGAGKTVSAKFVMRYLASVAASKTRNGG-----------  116
DdMyo1E        NDAYRSMRQSQENQCVIISGESGAGKTEASKKIMQFLTFVSSNQSPNG------------  111
HsMhc2         DNAYQFMLTDRENQSILITGESGAGKTVNTKRVIQYFATIAVTGEKKKE-----------  112
OcMhc1         DNAYQFMLTDRENQSILITGESGAGKTVNTKRVIQYFATIAVTGDKKKE-----------  112
                 .:  :     .*.::::********   *  : .::

                                                           Switch-1
CaMyo51A       ASSVASSALSTTGSASAPANSEIVQRVLDSNPLLEAFGNAKTLRNDNSSRFGKYIQLQFD  239
CaMyo51D       ------------SEID---ADAVVNRILDSNPLLEAFGNAKTRRNDNSSRFGKYIVLQFD  165
CaMyo51B       ------------ADGGAKFSDPIVQRIMESNPLLEAFGNAKTRRNDNSSRFGKYTQLQFD  168
CaMyo51C       ------------GGGN----NVVVNRVLESNPLLEAFGNAKTRRNDNSSRFGKYCQLQFD  164
CeMyo5         ------------T-------TSIEARVLASNPIMESIGNAKTIRNDNSSRFGKFIQINFC  157
DdMyo1E        --------------------ERISKMLLDSNPLLEAFGNAKTLRNDNSSRFGKYMEMQFN  151
HsMhc2         ------------EITSGKIQGTLEDQIISANPLLEAFGNAKTVRNDNSSRFGKFIRIHFG  160
OcMhc1         ------------EATSGKMQGTLEDQIISANPLLEAFANAKTVRNDNSSRFGKFIRIHFG  160
                                     :   :: :**::*::.**** **********:  ::*

CaMyo51A       NPLSHQLHNASSIDDMAVAATGMSSRQQVLAQSTGTASRCILAGSKCEVYLLEKSRVISH  299
CaMyo51D       RKEPKN---------------------------VGDNPRAIMSGSKCEVYLLEKSRVVYH  198
CaMyo51B       MGDKSS----------------------KAFATDKASRKCNLAGSKCEVYLLEKNRVTTH  206
CaMyo51C       MGTKAE----------------------QEFRG-RDKATCVLAGSKCDVYLLEKNRVVMH  201
CeMyo5         ERGR------------------------------------RIVGAEMKTYLLEKSRLVFQ  181
DdMyo1E        AVGS-------------------------------------PIGGKITNYLLEKSRVVGR  174
HsMhc2         TTGK-------------------------------------LASADIETYLLEKSRVVFQ  183
OcMhc1         TTGK-------------------------------------LASADIETYLLEKSRVTFQ  183
                                                        ...   *****.*:  :

CaMyo51A       AEGERTFHIFYQLLAAPEEYKATVWSGLEDTDNESFCYVGWTDTNTIEGRSDEDRFQLTL  359
CaMyo51D       DETERTYHIFYQLLNAPESEKVKIWKGLKGTKNESFAYVGKSPEVRIDGKEDSEHFPETK  258
CaMyo51B       DTTERTYHSFYQLLAAPENEKVEIWSELKGTNYESFAYIGTPPTDVIEGKKDGDHWHTTL  266
CaMyo51C       DPEERTYHIFYQIIASK--HKTKYWSHLAGTTNESFRFVGKAPTDTIEGMKDGDHFNETV  259
CeMyo5         APGERNYHIFYQLCAARNHQVLKD--LHLGPC-ESYSYLTQGGDSRIPGVDDKADFEALL  238
DdMyo1E        TQGERSFHIFYQMLKGLSQSKLNE--LGLTPNAPAYEYLKKSGCFDVSTIDDSGEFKIIV  232
HsMhc2         LKAERSYHIFYQITSNKKPELIEM--LLITTNPYDYPFV-SQGEISVASIDDQEELMATD  240
OcMhc1         LKAERSYHIFYQIMSNKKPDLIEM--LLITTNPYDYAFV-SQGEITVPSIDDQEELMATD  240
                  **.:* ***:                      : ::       :   .*

CaMyo51A       NSLALVGVIDDKLKALLRAICIVLQLGNLEFAP--LDD----DKCVISTDEELMELAELM  413
CaMyo51D       KALEIVGIKDDKYITLFQAIAAVLQLGNLSFAPKPGND----EESVVTSKKEFKELAELI  314
CaMyo51B       EILDTINVKGNDRRDLMRAMCIVLQLGNVSFMKDPNDD----DRSVVKDRKEFDKLAALM  322
CaMyo51C       KVLEKVGVKDDNLMTLMQAIIIVLQIGNLTFGPDPKDD----ERSVVTSTKDFAALSEIM  315
CeMyo5         KALQLLGFDEKQMSDVFRLLAGLLLLGNVHFENGEGSSAVSASSC--QEISR--LCREFW  294
DdMyo1E        KAMETLGLKESDQNSIWRILAAILHIGNITFAEAAEQRTGTTT-VKVSDTKSLAAAASCL  291
HsMhc2         SAIDILGFTNEEKVSIYKLTGAVMHYGNLKFKQKQREEQAEPDGTEVAD-----KAAYLQ  295
OcMhc1         SAIDILGFTSDERVSIYKLTGAVMHYGNMKFKQKQREEQAEPDGTEVAD-----KAAYLQ  295
               . :  :..  ..   : :    ::  **: *     .
```

```
CaMyo51A        DVPPDELAVALLVRTVTAKGE----TFQVPLKAETAKDSTDAFAKEIYAKTFLWLVRTIN 469
CaMyo51D        GCEEDLLGRAFTERTIKTRGE----EYKVPLKSDVSKESADAFAKEIYAKIFLWLVRAIN 370
CaMyo51B        GVTNQDLVDCLTERTMKTRNE----SYKVPLNADVAKESADAFAKEIYSKLFLWLVRALN 378
CaMyo51C        CVPEEAMECSFTERTMKTRNE----TYKVPLNATVAKESAEAFAKEIYGRLFLWLVRAIN 371
CeMyo5          KISESDLRIWLTRREIRAVNE----IVTKPLTKNEAVRSRDALTKMLYSHLFGWLVDKIN 350
DdMyo1E         KTDQQSLSIALCYRSISTGVGKRCSVISVPMDCNQAAYSRDALAKALYERLFNWLVSKIN 351
HsMhc2          SLNSADLLKALCYPRVKVGNEYVT----KGQTVEQVSNAVGALAKAVYEKMFLWMVARIN 351
OcMhc1          SLNSXDLLKALCYPRVKVGNEYVT----KGQTVQQVYNAVGALAKAVYEKMFLWMVTRIN 351
                      :  :     : .                    :  *::* :* : * *:*  :*

                                      Switch-2
                                      ┌────────┐
CaMyo51A        DATCAENNYRNASFDHQRFGVIGLLDIFGFETFEVNRFEQLCINYANEKLQQKFTQDIFR 529
CaMyo51D        DATCAENNYPGADAN-TEFGVVGLLDIFGFESFPINGFEQLCINYCNEKLQAKFTHDIFQ 429
CaMyo51B        DATCAELNYEGG-MD-KHFGVIGLLDIFGFESFVRNRFEQLCINYANEKLQAKFTEDIFR 436
CaMyo51C        DATSAELNYKSG-LM-EHFGVIGLLDIFGFESFVRNRFEQLCINYANEKLQAKFTEDIFR 429
CeMyo5          EALNEKDKLDGTNQKKRPDRFIGVLDIYGFETFDVNSFEQFSINYANEKLQQQFNQHVFK 410
DdMyo1E         TIINCT---------TEKGPVIGILDIYGFEVFQNNSFEQLNINFCNEKLQQLFIELTLK 402
HsMhc2          QQLDTK---------QPRQYFIGVLDIAGFEIFDFNSLEQLCINFTNEKLQQFFNHHMFV 402
OcMhc1          QQLDTK---------QPRQYFIGVLDIAGFEIFDFNSLEQLCINFTNEKLQQFFNHHMFV 402
                                    .:*:*** *** *  * :**: **: *****  * .  :

CaMyo51A        SVQTEYEFEGIELGEITYDDNTDV-LDLVEGGMGLLALLNEECVRPKGSDKTFVFKAQAM 588
CaMyo51D        TVQEEYKAEGLNLDDIQYDDNSDV-LELIEGKFGIIKQLNEECVRPKGNDEAFVSKALQS 488
CaMyo51B        SVQEEYEFEGIPLDEIKYDDNTDV-LDLIEGKAGLLAMLNEECVRPKGGDDAFVAKALAA 495
CaMyo51C        SVQDEYEKEGIPLDEIKYDDNTDV-LDLIEGKTGLLAMLNEECVRPKGTDQAFVTKALAA 488
CeMyo5          LEQEEYIREEIEWVRVDFHDNQPA-IDLIEGPVGMINLLDEQCKRLNGSDADWLSQLQNS 469
DdMyo1E         SEQEEYVREGIEWKNIEYFNNKPICELIEKKPIGLISLLDEACLIAKSTDQTFLDSICKQ 462
HsMhc2          LEQEEYKKEGIEWTFIDFGMDLAACIELIEKPMGIFSILEEECMFPKATDTSFKNKLYDQ 462
OcMhc1          LEQEEYKKEGIEWEFIDFGMDLAACIELIEKPMGIFSILEEECMFPKATDTSFKNKLYEQ 462
                  * **  * :    : :  :      : :    *::  *:* *   :.  *  :  .

CaMyo51A        NK-------DNAVWIREPHYSATSFGIRHYAGKVTYDSNGFVTKNMDTLPTDLQDCAAKS 641
CaMyo51D        NK-------TVPCLIAKNTFKRSEFGINHYAGAVIYTAQDFVIRNTDTLPTDLQVCAKAC 541
CaMyo51B        NK-------KSPCLIASK-TNRREFGIHHYAGKVMYDAEGFVGSNVDTLPTDLGECAAKS 547
CaMyo51C        NK-------QSPCLFPNK-VNRMGFGIHHYAGKVMYDAEEFVIRNQDTLPTDLSDLASLC 540
CeMyo5          TELKRNPQLAFPK------VRSNDFIVRHFAADVTYSTDGFVEKNRDAIGEQLLDVVVAS 523
DdMyo1E         FE-KN-PHLQSYVVSKDRSIGDTCFRLKHYAGDVTYDVRGFLDKNKDTLFGDLISSMQSS 520
HsMhc2          HL-GKSANFQKPK--VVKGKAEAHFALIHYAGVVDYNITGWLEKNKDPLNETVVGLYQKS 519
OcMhc1          HL-GKSNNFQKPK--PAKRKVEAHFSLVHYAGTVDYNITGWLDKNKDPLNETVVGLYQKS 519
                                        * : *:*. * *    ::  * * :   :      .

CaMyo51A        SNAILAKELTNEAMMNPFAPVQSSTKAPETAAAAAAPKKKKKRPKGARGRPGGAPTASSA 701
CaMyo51D        KNEILAKHLDNDKCTNQESK------AP----------------AP-------------- 565
CaMyo51B        NNEIICKHLNNDKSTNLTKA------P-----------------T--------------- 569
CaMyo51C        SNEIIAKHMTNDSCTNFESK------T-----------------KS-------------- 563
CeMyo5          KFPFIRTVIGSTAPTSVSS----------------------------------------- 542
DdMyo1E         SDPLVQGLFPPTRPEDS------------------------------------------- 537
HsMhc2          AMKTLAQLFSGAQTAEGEG----------------------------------------- 538
OcMhc1          AMKTLAFLFTGTAAAEAE------------------------------------------ 537
                    :   :      .

                                                              actin binding
                                                               ┌──────────┐
CaMyo51A        SAPTSRPGHHMPRRSVKSNLVAETVWTKFKTQLSRLMTQLNDTRTRYIRCIKPNSKKRPR 761
CaMyo51D        ---AA-KSGRGPPRRAKSNLVADTVMTKFKTQLSSLMKGLSTTKSRYIRCVKPNVLKKKL 621
CaMyo51B        ----P-GAAKKAPRRAKSNLVAATVWTKYKSQLMSLMNMLAQTNSRYIRCIKPNTYKKPS 624
CaMyo51C        ---AQ-VAKKAPPKRAKSNLVAATVWAKYKTQLMSLMNMLKTTESKYIRCIKPNAPKKPL 619
CeMyo5          SSSSSTP---------GKRTIKKTVASQFRDSLKELMSVLCSTRPHYVRCIKPNDSKISF 593
DdMyo1E         -----KK-------------RPETAGSQFRNAMNALITTLLACSPHYVRCIKSNDNKQAG 579
HsMhc2          AGGGAKKGG------KKKGSSFQTVSALFRENLNKLMTNLRSTHPHFVRCIIPNETKTPG 592
OcMhc1          --GGGKKGG------KKKGSSFQTVSALFRENLNKLMTNLRTHSPHFVRSIIPNETKTPG 589
                                       *. :  ::  :  *:. *      :::*.:  *  *
```

Supplementary Figure 9: Sequence alignment of the motor domain of the *C. australis* myosins CaMyo51A-D and myosins from *Oryctolagus cuniculus* (Genbank ID AAA74199.1); *Homo sapiens* (Gene bank ID: AAD29950.1), *Caenorhabditis elegans* (Genbank ID: AAA97926.1), *Dictyostelium discoideum* (Genbank ID: EAL63071.1). Identical amino acids are indicated by (*) and highlighted in grey, a : (colon) indicates conservation between groups of strongly similar properties; a . (period) indicates conservation between groups of weakly similar properties

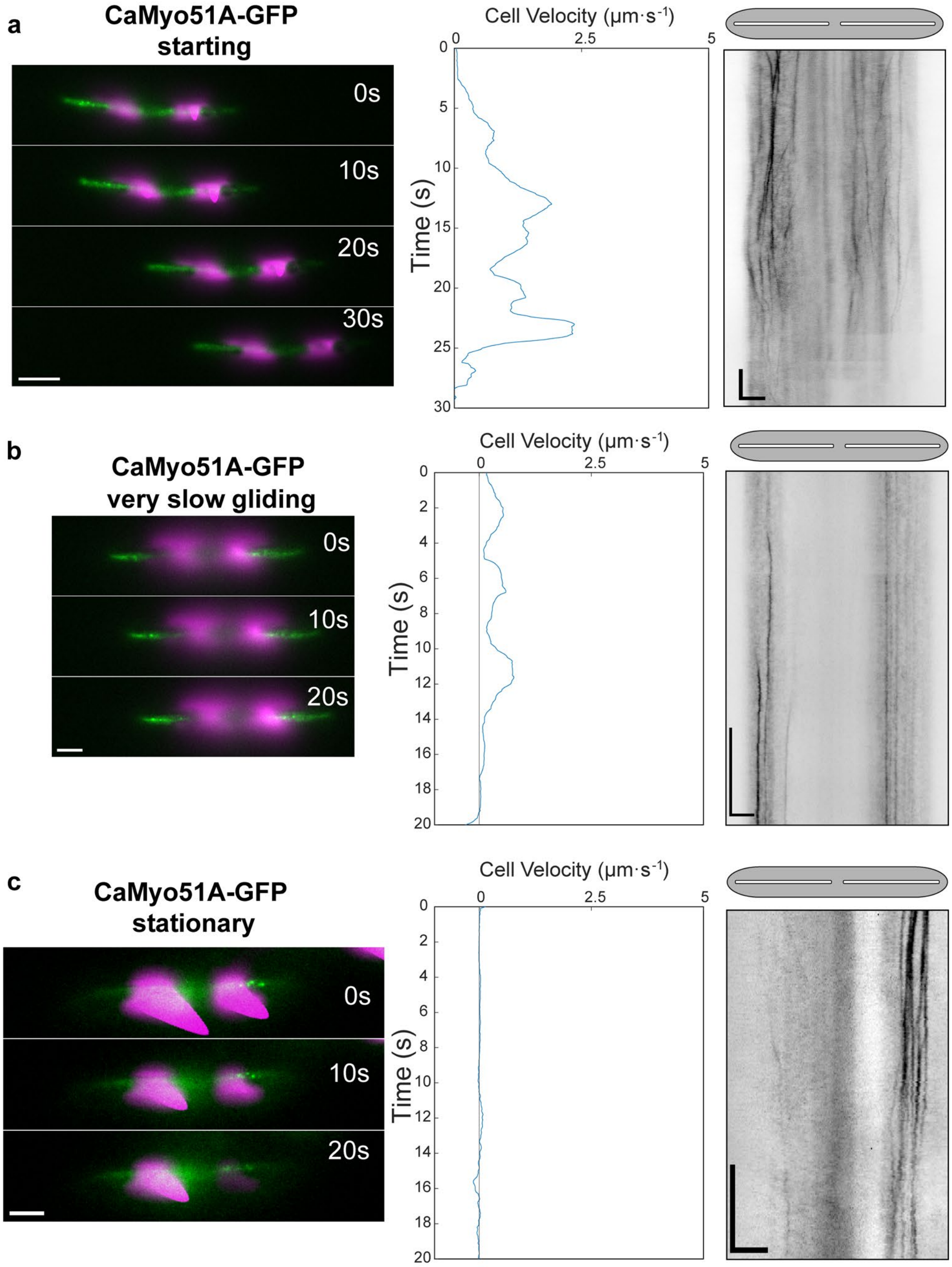

Supplementary Figure 10: Kymograph analysis of CaMyo51A-GFP in starting, slowly-moving and stationary cells. Analysis of 25-30 s time-lapse segments of CaMyo51A-GFP expressing cells upon **a** starting (Supplementary Movie S7), **b** slowly-moving (Supplementary Movie S8) and **c** being stationary (Supplementary Movie S9). (Left panels) Montages showing the position of cells at 10 s intervals (GFP in green, chloroplast autofluorescence in magenta, scale bars: 5 µm). (Middle panels) Cell velocity as function of time, generated from chloroplast tracking data. (Right panels) Registered kymographs generated from GFP-channel data (black) showing movement of myosins relative to the cell. Gray ellipses and white slits above kymographs approximate the positions of the cell body and raphe openings, respectively. (Scale bars: horizontal = 5 µm, vertical = 5 s)

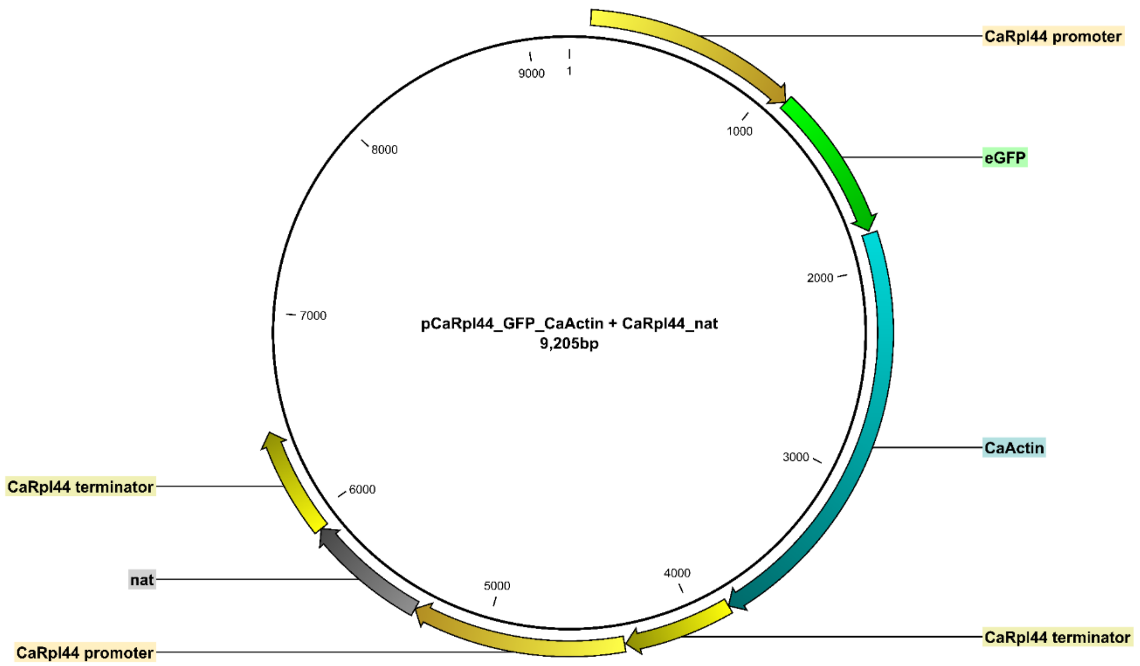

Supplementary Figure 11: Plasmid map of the DNA construct for N-terminal eGFP tagging of the *C. australis* Actin. nat = nourseothricin (ClonNat) resistance gene; eGFP = enhanced green fluorescent protein.

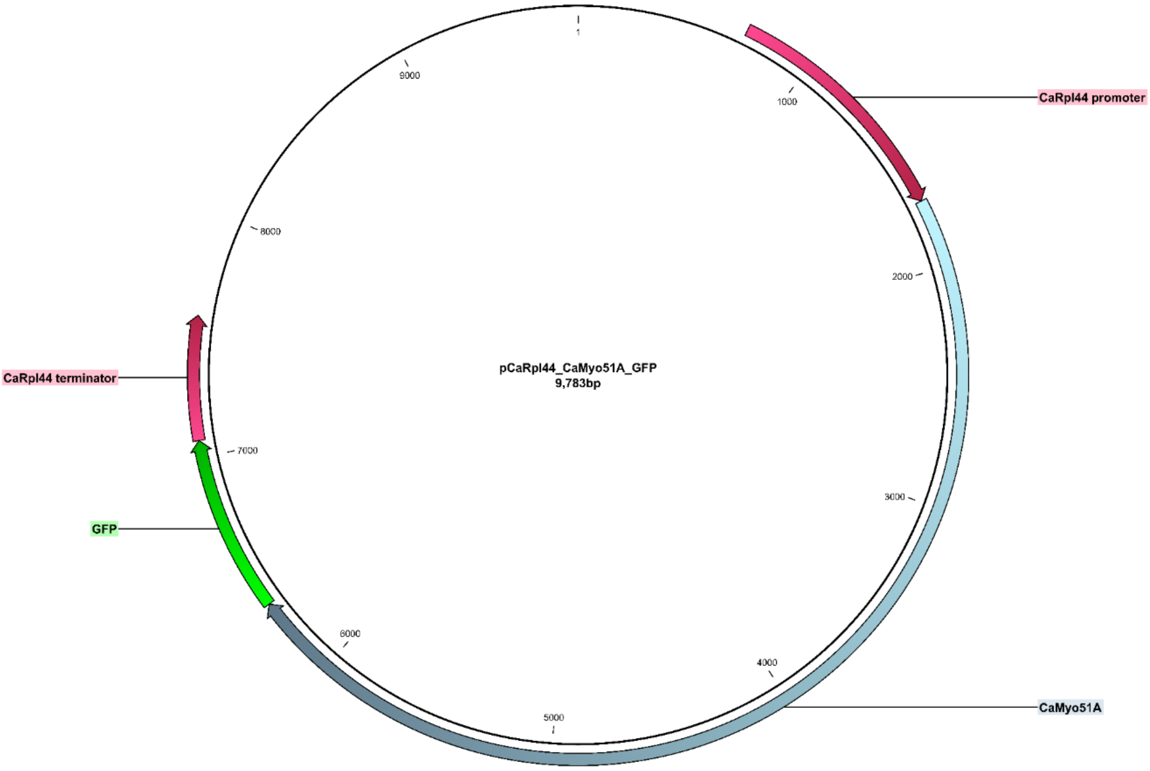

Supplementary Figure 12: Plasmid map of the DNA construct for C-terminal eGFP tagging of the *C. australis* CaMyo51A under the control of the CaRpl44 regulatory sequences. eGFP = enhanced green fluorescent protein.

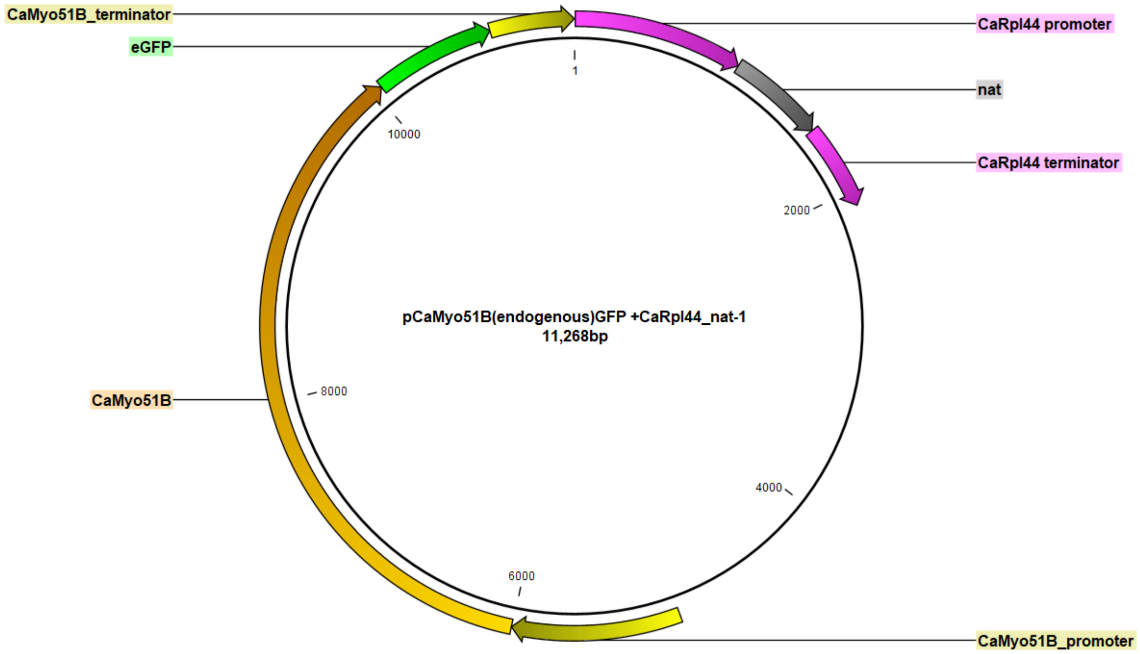

Supplementary Figure 13: Plasmid map of the DNA construct for C-terminal eGFP tagging of the *C. australis* CaMyo51B under the control of its endogenous CaMyo51B regulatory sequences. nat = nourseothricin (ClonNat) resistance gene; eGFP = enhanced green fluorescent protein.

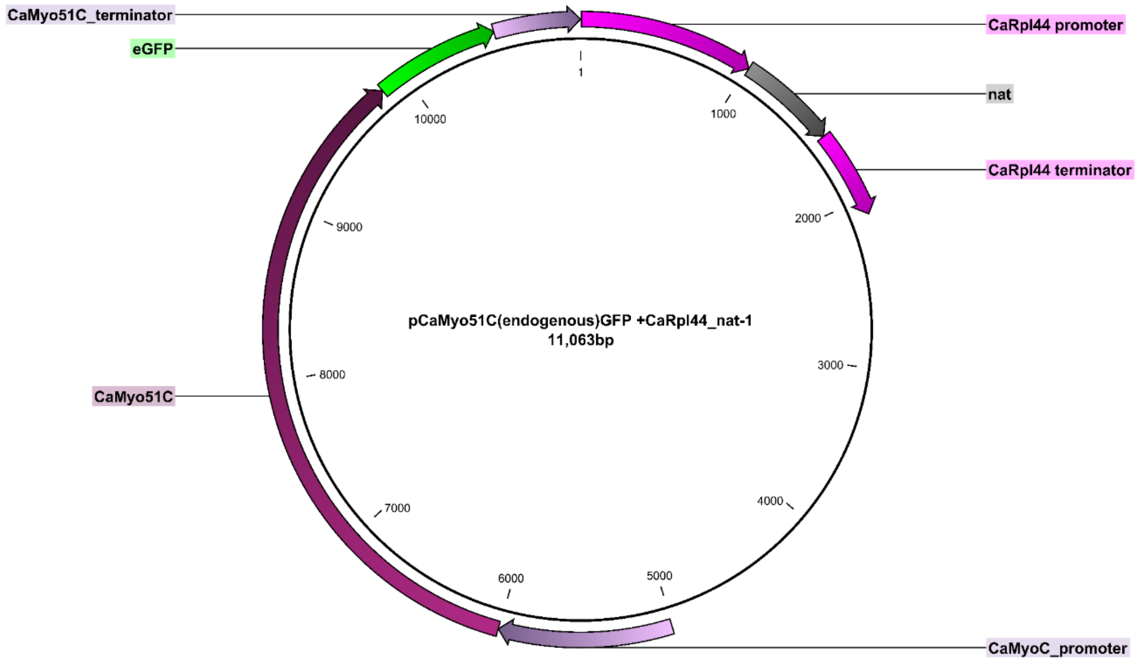

Supplementary Figure 14: Plasmid map of the DNA construct for C-terminal eGFP tagging of the *C. australis* CaMyo51C under the control of its endogenous CaMyo51B regulatory sequences. nat = nourseothricin (ClonNat) resistance gene; eGFP = enhanced green fluorescent protein.

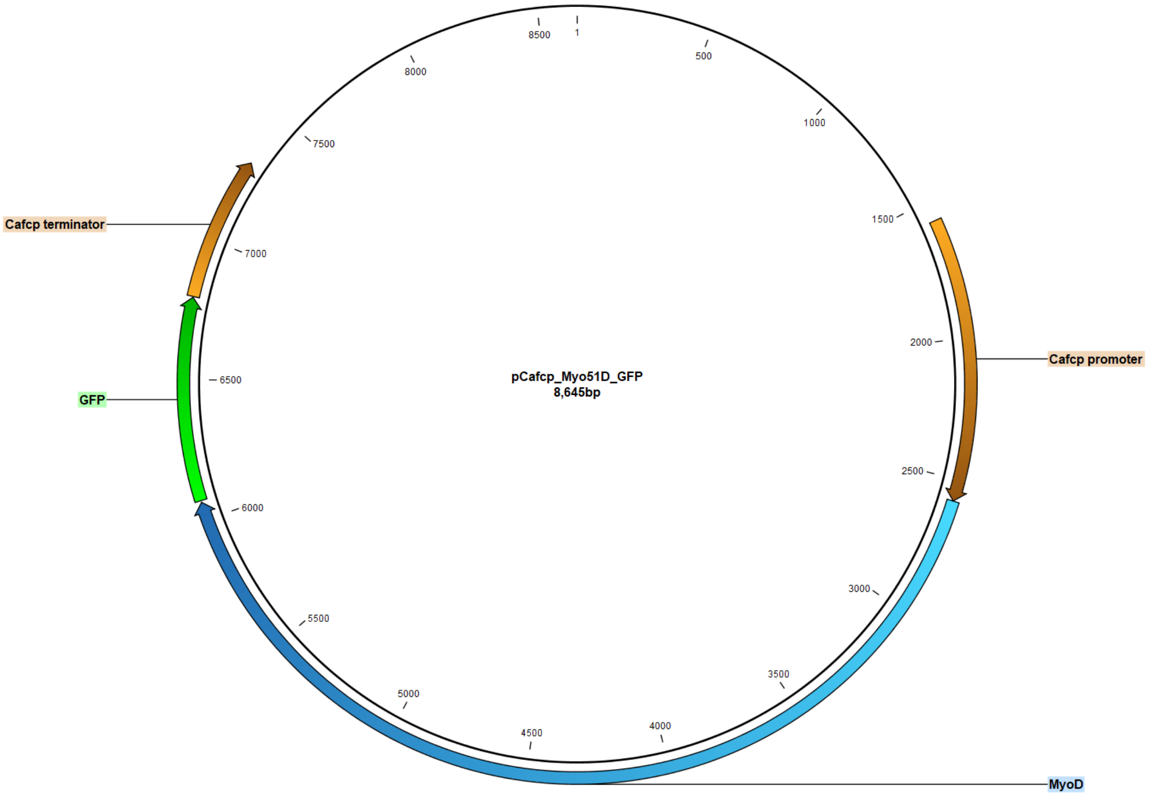

Supplementary Figure 15: Plasmid map of the DNA construct for C-terminal eGFP tagging of the *C. australis* CaMyo51D under the control of the Cafcp regulatory sequences. eGFP = enhanced green fluorescent protein.

Supplementary Tables

Supplementary Table 1: Primers used to determine full-length gene models

Gene	Sequence
**CaMyo51A**	
3'RACE	1ST PCR: 5'-AGGGTCCTCGATTCAAACCCGCTCCTCGAAGCA-3';<br>2nd PCR: 5'-GTGCGTCCGAAGGGCTCCGACAA-3'
5'RACE	1ST PCR: 5'-CAGGGTACCCTATTCGAAACCGAAGATGTCGAG-3';<br>2nd PCR: 5'- GCCAGC GCGGATGCTTCGTAGA-3'
Full-length gene model	sense: 5'-CCACAGACAGCGCTGCGACT-3'<br>antisense 5'-CAGGGTACCCTATTCGAAACCGAAGATGTCGAG-3'
Full-length gene model	sense: 5'-AGGGAATTCGATTCAAACCCGCTCCTCGAAGCA-3'<br>antisense: 5'-GGC GATGTAGGTTGCCTT-3'
**CaMyo51B**	
3'RACE	1st PCR: 5'-GCAGCCAGATCATCGCTTACC-3'<br>2nd PCR: 5'-CGCAAGGACTTCGACATCATG-3'
5' RACE	1st PCR: 5'-CCCTCGTGGTCCTCCCAGACG-3'<br>2nd PCR: 5'-GGGCAAGTTGACCATGTCCGG -3'
Full-length gene model	sense: 5'-AAACAACAATTACAACAACCTACATGGGCAAGAAAAAGGCC-3'<br>antisense: 5'- CCTCGCCCTTGCTCACCATTCTAGAAGCTACGGATTCGGCAGC-3'
**CaMyo51C**	
3'RACE	1st PCR: 5'-CCTCATCCAGGCCAACGAGTC-3'<br>2nd PCR: 5'-CGTTCGAGATGTTGAACAACC-3'
5' RACE	1st PCR: 5'-GCTCCACCATGTCTGGGAAGG-3'<br>2nd PCR: 5'-ACTGTGGCACGCGGACTTGGG-3'
Full-length gene model	sense: 5'-AAACAACAATTACAACAACCTACGTATGGGCGAGAAAAAGTCCCAATACG-3'; antisense: 5'-CCTCGCCCTTGCTCACCATTCTAGACAGGCCTGGGGCGGATGC-3'
**CaMyo51D**	
3'RACE	1ST PCR: 5'-TGCGAGAGCATGAAGCGTGAC-3'<br>2nd PCR: 5'-CGCGAAGGGATTGAACGCAAC-3'
5' RACE	1st PCR: 5'-GATACGGTTGACGACCGCATC-3'<br>2nd PCR: 5'- GTCGCCGACGTTCTTGGGCTC-3'
Full-length gene model	sense: 5'-AAACAACAATTACAACAACCTACATGCCAAAGGAAAAGGAC-3'<br>antisense: 5'- CCTCGCCCTTGCTCACCATTCTAGAATCGGAATCAGAGTCCGAG-3'
**CaMyo51E**	
3'-end	manual annotation
5' RACE	1st PCR: 5'-GATACCTGTTGCTTCCTTTTG-3'

	2nd PCR: 5'-TCGCAGCAGCGCTCTTGTTGC-3'
**CaMyo52**	
3'RACE	1st PCR: 5'-CAGGAATGCAAACGACTT-3'<br>2nd PCR: 5'-CGCGTTGCACCAATATCA-3'
5' RACE	1st PCR: 5'-TTGTCTACCTGCTCTTCCG-3'<br>2nd PCR: 5'-GCACGACATTGCCATCCTC-3'
**CaMyo53**	
3'RACE	1st PCR: 5'-CAGCAACAACAGCAGCAA-3'<br>2nd PCR: 5'-GAGAACGGAGTGCCAAAG-3'
5' RACE	1st PCR: 5'-ATTTCTGGTTCTCCAAGC-3'<br>2nd PCR: 5'-GCTTGAAGGGGTTGATGG-3'
**CaMyo47**	
3'RACE	1st PCR: 5'-GGCATTTTGGGACGACAT-3'<br>2nd PCR: 5'-CGATATTTTGCCGTCACT-3'
5' RACE	1st PCR: 5'-GAAACCAGGATCGATTGG-3'<br>2nd PCR: 5'-GCATCGTAGCTGAGGGAGA-3'
**CaMyoI**	
3'RACE	1st PCR: 5'-CGTCGATGAGGATGAGAT-3'<br>2nd PCR: 5'-CGAAGATGTGAAGGCCAA-3'
5' RACE	1st PCR: 5'-GGATGGATTGAGATTTGC-3'<br>2nd PCR: 5'-CGATGGATGAAAGATGCC-3'
**CaMyoJ**	
3'RACE	1st PCR: 5'-CAAGGAAGGCAAGGAGCA-3'<br>2nd PCR: 5'- GTCATGAATGGGGCAACC -3'
5' RACE	1st PCR: 5'- AGAATGGCCTGGTTGCAG -3'<br>2nd PCR: 5'- CTTGTCCCAGTAGCGCTT -3'
**CaMyo29**	
3'RACE	1st PCR: 5'-ATGAGCCGCGTGTCCATGAAC-3'<br>2nd PCR: 5'-GTCTCAAGAAGGCCTTCTGCG-3'
5'-end	truncated, manual annotation
**CaMyoL**	
3'RACE	1st PCR: 5'-CAACGATTTGCGAGAGGG-3'<br>2nd PCR: GAAACAGTCAAGGCGAAG-3'
5' RACE	1st PCR: 5'-GATTGGTTGACTTGCACG-3'<br>2nd PCR: 5'-TGGAGATGGGTCAAGGTG-3'

References

1. Zackova Suchanova, J. *et al.* Diatom adhesive trail proteins acquired by horizontal gene transfer from bacteria serve as primers for marine biofilm formation. *New Phytol.* **240**, 770–783 (2023).

2. Priyam, A. *et al*, Sequenceserver: A Modern Graphical User Interface for Custom BLAST Databases, *Molecular Biology and Evolution*, **36**, 2922–2924(2019).

3. Keeling PJ, Burki F, Wilcox HM, Allam B, Allen EE, et al.The Marine Microbial Eukaryote Transcriptome Sequencing Project (MMETSP): Illuminating the Functional Diversity of Eukaryotic Life in the Oceans through Transcriptome Sequencing. *PLOS Biology* **12,** e1001889. (2014)

4. Osuna-Cruz, C.M., Bilcke, G., Vancaester, E. *et al.* The *Seminavis robusta* genome provides insights into the evolutionary adaptations of benthic diatoms. *Nat Commun* **11**, 3320 (2020).
