# Supplementary material for: Gliding motility of the diatom Craspedostauros australis coincides with the intracellular movement of raphid-specific myosins
Source: Commun Biol. 2024 Sep 23;7:1187. doi: 10.1038/s42003-024-06889-w (PMC11420354; doi:10.1038/s42003-024-06889-w)
Supplement: Supplementary file 3 — Description of Additional Supplementary Files [file 42003_2024_6889_MOESM3_ESM.pdf]

Description of Additional Supplementary Files

File name: Supplementary Movie 1

Description: Gliding GFP-actin expressing cell imaged by TIRF microscopy. Movie show composites of GFP and chloroplast channels (upper panel), and registered movies of only the GFP channel data (lower panel). Scale bars: 5 µm.

File name: Supplementary Movie 2

Description: A stationary GFP-actin expressing cell imaged by TIRF microscopy. Movie show composites of GFP and chloroplast channels (upper panel), and registered movies of only the GFP channel data (lower panel). Scale bars: 5 µm.

File name: Supplementary Movie 3

Description: Example of a CaMyo51A-GFP expressing cell exhibiting smooth, sustained gliding imaged by TIRF microscopy. Movie show composites of GFP and chloroplast channels (upper panel), and registered movies of only the GFP channel data (lower panel). Scale bars: 5 µm.

File name: Supplementary Movie 4

Description: Example of a CaMyo51B-GFP expressing cell exhibiting smooth, sustained gliding imaged by TIRF microscopy. Movie show composites of GFP and chloroplast channels (upper panel), and registered movies of only the GFP channel data (lower panel). Scale bars: 5 µm.

File name: Supplementary Movie 5

Description: Example of a CaMyo51C-GFP expressing cell exhibiting smooth, sustained gliding imaged by TIRF microscopy. Movie show composites of GFP and chloroplast channels (upper panel), and registered movies of only the GFP channel data (lower panel). Scale bars: 5 µm.

File name: Supplementary Movie 6

Description: Example of a CaMyo51D-GFP expressing cell exhibiting smooth, sustained gliding imaged by TIRF microscopy. Movie show composites of GFP and chloroplast channels (upper panel), and registered movies of only the GFP channel data (lower panel). Scale bars: 5 µm.

File name: Supplementary Movie 7

Description: TIRF microscopy imaging of a CaMyo51A-GFP expressing cell transitioning from stationary to gliding of. Movie show composites of GFP and chloroplast channels (upper panel), and registered movies of only the GFP channel data (lower panel). Scale bars: 5 µm.

File name: Supplementary Movie 8

Description: TIRF microscopy imaging of a slow moving CaMyo51A-GFP expressing cell. Movie show composites of GFP and chloroplast channels (upper panel), and registered movies of only the GFP channel data (lower panel). Scale bars: 5 µm.

File name: Supplementary Movie 9

Description: TIRF microscopy imaging of a stationary CaMyo51A-GFP expressing cell. Movie show composites of GFP and chloroplast channels (upper panel), and registered movies of only the GFP channel data (lower panel). Scale bars: 5 µm.

File name: Supplementary Movie 10

Description: TIRF microscopy imaging of a CaMyo51B-GFP expressing cell transitioning from stationary to gliding. Movie show composites of GFP and chloroplast channels (upper panel), and registered movies of only the GFP channel data (lower panel). Scale bars: 5 µm.

File name: Supplementary Movie 11

Description: TIRF microscopy imaging of a CaMyo51B-GFP expressing cell transitioning from gliding to stationary. Movie show composites of GFP and chloroplast channels (upper panel), and registered movies of only the GFP channel data (lower panel). Scale bars: 5 µm.

File name: Supplementary Movie 12

Description: TIRF microscopy imaging of reversal event of a CaMyo51B-GFP expressing cell. Movie show composites of GFP and chloroplast channels (upper panel), and registered movies of only the GFP channel data (lower panel). Scale bars: 5 µm.

File name: Supplementary Movie 13

Description: TIRF microscopy imaging of a CaMyo51C-GFP movement from the cell apices to center of cell. Movie show composites of GFP and chloroplast channels (upper panel), and registered movies of only the GFP channel data (lower panel). Scale bars: 5 µm.

File name: Supplementary Movie 14

Description: TIRF microscopy imaging of CaMyo51D-GFP movement from the cell apices to center of cell. Movie show composites of GFP and chloroplast channels (upper panel), and registered movies of only the GFP channel data (lower panel). Scale bars: 5 µm.

File name: Supplementary Movie 15

Description: TIRF microscopy imaging of CaMyo51D-GFP movement from the center to apices of cell. Movie show composites of GFP and chloroplast channels (upper panel), and registered movies of only the GFP channel data (lower panel). Scale bars: 5 µm.

File name: Supplementary Data 1

Description: List of the names and abbreviations of the diatom myosins used to construct the Cladogram in Supplementary Fig. 4. The databases used to retrieve the sequences are: MMTSP (http://sampgr.org.cn/sequenceserver/)[2,3] ; PLAZA (https://bioinformatics.psb.ugent.be/plaza/versions/plaza_diatoms_01/)[4];

File name: Supplementary Data 2

Description: Clustal Omega multiple sequence alignment of diatom myosin sequences used for the phylogenomic analysis (Supplementary Figure 7)

File name: Supplementary Data 3

Description: Source data for the analysis of the relationship between cell velocity and intracellular GFP-tagged myosin velocities presented in Figure 4.
